# Asymmetric Synthesis of (−)-6-Desmethyl-Fluvirucinine A_1_ via Conformationally-Controlled Diastereoselective Lactam-Ring Expansions

**DOI:** 10.3390/molecules23092351

**Published:** 2018-09-14

**Authors:** Hyunyoung Moon, Hojong Yoon, Changjin Lim, Jaebong Jang, Jong-Jae Yi, Jae Kyun Lee, Jeeyeon Lee, Younghwa Na, Woo Sung Son, Seok-Ho Kim, Young-Ger Suh

**Affiliations:** 1 Department of Pharmacy, College of Pharmacy and Institute of Pharmaceutical Sciences, CHA University, 120 Haeryong-ro, Pocheon 11160, Gyeonggi-do, Korea; hyunyoungmoon@gmail.com (H.M.); koryoi0709@gmail.com (C.L.); nmryi222@gmail.com (J.-J.Y.); yna7315@cha.ac.kr (Y.N.); wson@cha.ac.kr (W.S.S.); 2 College of Pharmacy, Seoul National University, 1 Gwanak-ro, Gwanak-gu, Seoul 08826, Korea; hojong_yoon@g.harvard.edu (H.Y.); jaebong.jang@gmail.com (J.J.); jyleeut@snu.ac.kr (J.L.); 3 Center for Neuro-Medicine, Korea Institute of Science and Technology (KIST), Seoul 02792, Korea; j9601@kist.re.kr

**Keywords:** fluvirucinine, aza-Claisen rearrangement, amidoalkylation

## Abstract

The versatile synthesis of (−)-6-desmethyl-fluvirucinine A_1_ was accomplished at a 24% overall yield through a thirteen-step process from a known vinylpiperidine. The key part involved the elaboration of the distal stereocenters and a macrolactam skeleton via conformationally-induced diastereocontrol and the iterative aza-Claisen rearrangements of lactam precursors.

## 1. Introduction

**Figure 1:**
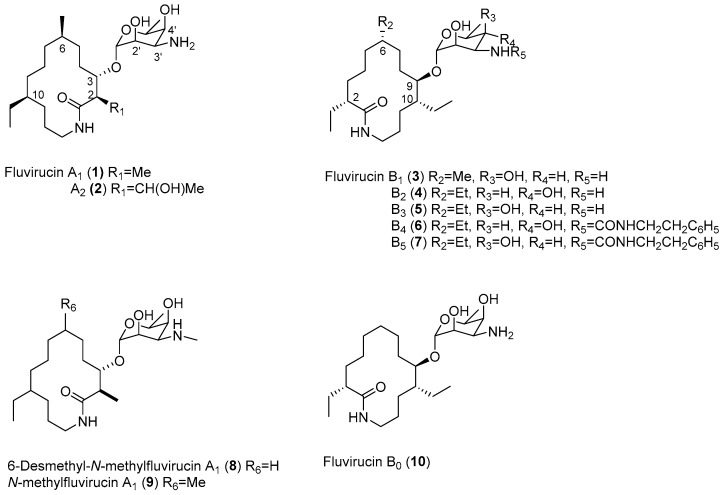
Structures of fluvirucins.

Fluvirucins, a class of macrolactam alkaloids, including fluvirucin A_1-2_ and B_1-5_ (**1**–**7**) (Figure 1) were isolated in the 1990s [1,2,3,4,5,6]. These macrolactam antibiotics have drawn significant attention due to their considerable inhibitory activities against the influenza A virus in Madin—Darby canine kidney (MDCK) cells [3,4]. They commonly consist of 2,6-dialkyl-10-ethyl-3(or 9)-hydroxy-13-tridecanelactam as an aglycon called fluvirucinine, which possesses four stereogenic centers and is connected to a carbohydrate by a glycosidic linkage. The synthesis of fluvirucinines has continuously attracted the attention of organic chemists [7,8,9,10,11,12,13,14,15] due to the difficult stereocontrol during the creation of distant stereogenic centers. Recently, three new macrolactams, including 6-desmethyl-*N*-methylfluvirucin A_1_ (**8**), *N*-methylfluvirucin A_1_ (**9**), and fluvirucin B_0_ (**10**), were isolated from *Nonomuraea terkmeniaca* MA7364 [16] and *Nonomuraea terkmeniaca* MA7381 [17], respectively. In particular, 6-desmethyl-*N*-methylfluvirucin A_1_ (**8**) exhibited in vitro activity (EC_90_ 15 ± 5 μg/mL) against *Haemonchus contortus* larvae. The absolute configurations of C_2_, C_3_, and C_10_ of **8** and **9** have not been determined yet, although the relative stereochemistry of C_2_ and C_3_ has been disclosed. The stereochemistries of C_2_, C_3_, and C_10_ of **8** and **9** have been considered the same as those reported for the fluvirucin A series [16].

**Figure 2:**
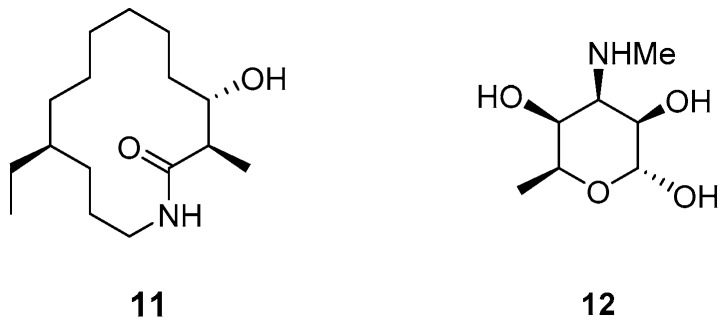
6-Desmethyl-fluvirucinine A_1_ (**11**) and the carbohydrate part (**12**) of 6-desmethyl-*N*-methylfluvirucin A_1_ (**8**).

Recently, we have been interested in 6-desmethyl-*N*-methylfluvirucin A_1_ (**8**) since it has biological activities even though 6-desmethyl-fluvirucinine A_1_ (**11**), which is an aglycon of **8**, is devoid of the characteristic C_6_-alkyl substituent of the fluvirucin family [16]. In particular, our interests are focused on the effect of the stereochemistry of 6-desmethyl-fluvirucinine A_1_ on biological activities (Figure 2). Along this line, we have been working on the synthesis of **13** as an antipode of 6-desmethyl-fluvirucinine A_1_ (**11**) [16]. Herein, we describe synthesis and structural confirmation of (−)-6-desmethyl-fluvirucinine A_1_ (**13**).

## 2. Results and Discussion

### 2.1. Synthetic Strategy for (−)-6-Desmethyl-Fluvirucinine A_1_ (***13***)

**Scheme 1:**
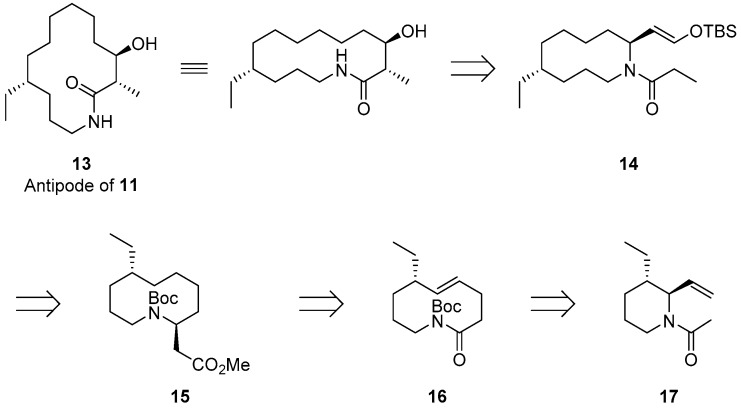
Retrosynthetic analysis for synthesis of **13** as an antipode of **11**.

Our synthetic strategy for **13** was based on the amide enolate-induced lactam ring expansion strategy [18,19,20], which was established by us for the synthesis of macrolactam alkaloids (Scheme 1) [14,15,21,22,23,24,25,26,27,28,29]. However, the diastereoselective aza-Claisen rearrangement (ACR) of **14**, which does not possess the characteristic C_6_-methyl substituent, remains a formidable task because the C_6_-substituent seemed to influence the formation of a chair-like transition state in the key ACR [14,15].

### 2.2. The First ACR and Diastereoselective Amidoalkylation for Synthesis of Ester ***15***

**Scheme 2:**
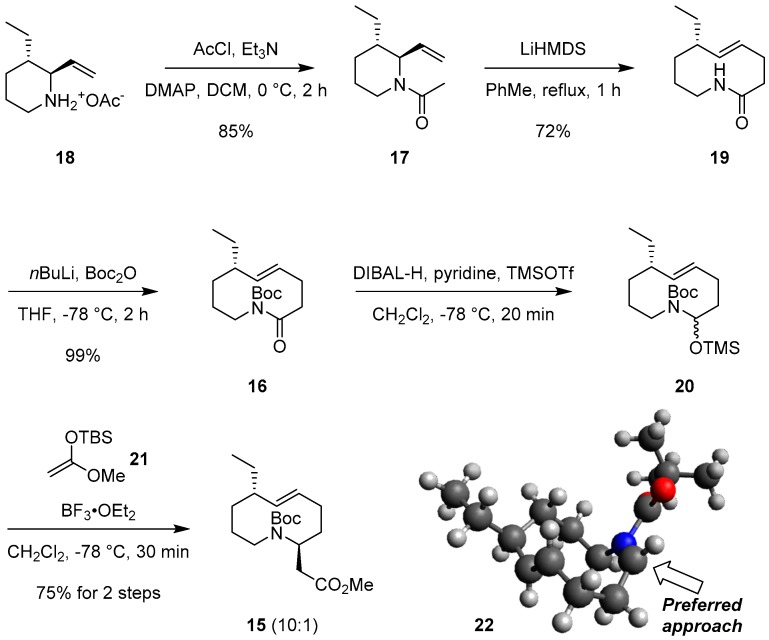
Preparation and diastereoselective amidoalkylation of **16**.

Our synthesis was commenced with the preparation of azacycle **17**, which is the first ACR precursor, through the acetylation of the known and optically active vinylpiperidine **18 [14]**, as shown in Scheme 2. The subjection of **17** to the first ACR (LiHMDS, toluene, reflux) [14,15,21,22,23,24] produced the ring-expanded lactam **19** with a 72% yield. Our initial attempt for the amidoallylation of **19**, which was commonly utilized to prepare a second ACR precursor in our previous syntheses [15,23,30,31,32,33], was not successful. We encountered difficult diastereocontrol in the amidoallylation of **19**, which was likely due to the absence of the 2-methyl substituent [15]. We anticipated that the ring-olefin in a medium sized-lactam system can induce an intrinsic ring strain that results in an improved diastereoselective amidoalkylation. In addition, we decided to execute an amidoalkylation with ketene acetal **21** as a bulky nucleophile [34]. After the Boc-protection of lactam **19**, the resulting lactam **16** was subjected to a sequence [15,23,30,31,32,33,34] of DIBAL-H reduction followed by the trapping of the resulting alkoxide with TMSOTf and the addition of ketene acetal **21** to the unstable *N*,*O*-acetal TMS-ether **20** in the presence of BF_3_∙OEt_2_. Indeed, a highly diastereoselective amidoalkylation of **16** was observed, which afforded methyl ester **15** with a 75% yield for three steps and a small amount of diastereoisomer (10:1). The excellent diastereoselectivity was likely due to the sterically favored *Si*-face attack of the bulky ketene acetal **21** in the energetically favorable (*Z*)-*N*-acyl iminium intermediate **22** that was generated from *N*,*O*-acetal TMS ether **20** [34,35].

### 2.3. The Second ACR and Completion of the Synthesis

**Scheme 3:**
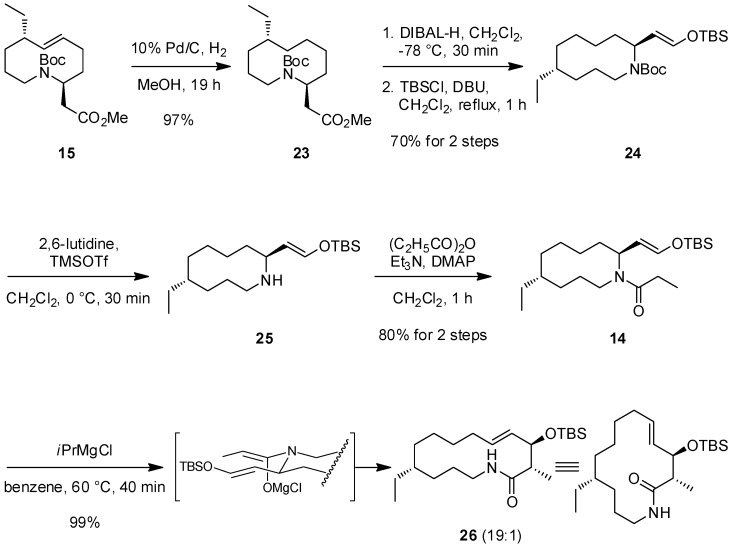
Synthesis of macrolactam **26**.

**Figure 3:**
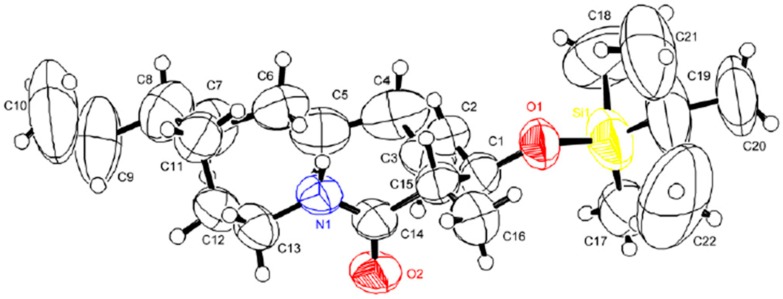
X-ray crystallographic structure of **32**. Displacement ellipsoids are drawn at the 50% probability level and H atoms are shown as small spheres of arbitrary radii; black = carbon, red = oxygen, blue = nitrogen, and yellow = silicon.

For the preparation of the second ACR precursor, olefin hydrogenation of **15** produced ester **23** as show in Scheme 3. DIBAL-H reduction of **23** and treatment of the resulting aldehyde with TBSCl in the presence of DBU in dichloromethane [36,37] selectively produced (*E*)-enol ether **24** with a 67% yield for three steps. Boc deprotection of **24** and propionylation of the resulting amine **25** afforded the second ACR precursor **14** with a 80% yield for three steps. Finally, subjection of **14** to the standard ACR conditions (*i*PrMgCl in benzene, 60 °C) [25,26,27,28,29] produced the desired ring-expansion product **26** with a 99% yield in favor of the *anti*-stereoisomer (19:1). The ACR with other bases, including LiHMDS, resulted in a low diastereoselectivity (≈1.1–1.2:1) for the *anti*-product. It is noteworthy that the *anti*-stereoisomer **26** in the absence of the 6-methyl substituent, which was considered important for the diastereoselective ACR, was selectively produced [15,23]. The stereochemistry of **26** was confirmed by X-ray crystallographic analysis (Figure 3) [38].

**Scheme 4:**
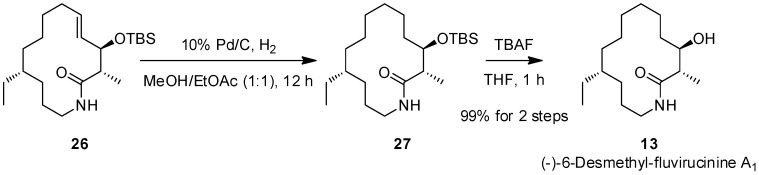
Completion of the (−)-6-demethyl-fluvirucinine A_1_ (**13**) synthesis.

For the completion of the synthesis, macrolactam **26** was hydrogenated and then desilylated to produce **14** with a 99% yield for two steps (Scheme 4).

## 3. Materials and Methods 

### 3.1. General Information

Unless stated otherwise, all reactions were performed under an argon atmosphere with dry solvents under anhydrous conditions. Tetrahydrofuran (THF) and Et_2_O were distilled immediately before use in sodium benzophenone ketyl. Dichloromethane, chloroform, triethylamine, acetonitrile, and pyridine were freshly distilled from calcium hydride. All starting materials and reagents were obtained from commercial suppliers and were used without further purification, unless otherwise noted. Solvents for routine isolation of products and chromatography were reagent grade and glass distilled. Silica gel 60 (230–400 mesh, Merck, Kenilworth, NJ, USA) was used for flash column chromatography. Reaction progress was monitored by thin-layer chromatography (TLC), which was performed using 0.25 mm silica gel plates (Merck, Kenilworth, NJ, USA). Optical rotations were measured with a P-2000 digital polarimeter (JASCO, Easton, MD, USA) at ambient temperature using a 100 mm cell of 2 mL capacity. ^1^H- and ^13^C-NMR spectra were recorded on a JNM-LA 300 (JEOL, Tokyo, Japan), AVANCE-500 (Brucker, Billerica, MA, USA), AVANCE-400 (Brucker, Billerica, MA, USA), and JNM-ECA-600 (JEOL, Tokyo, Japan). ^1^H-NMR data were reported as follows: chemical shift (parts per million, δ), multiplicity (br, broad signal; s, singlet; d, doublet; t, triplet; q, quartet; quint, quintet; m, multiplet and/or multiple resonances), coupling constant in hertz (Hz), and number of protons. Infrared spectra were recorded on a JASCO FT-IR-4200 spectrometer and are reported in the frequency of absorption (cm^−1^). High resolution mass spectra (HR-MS) were obtained with a JMS-700 (JEOL, Tokyo, Japan) instrument and Q TOF 6530 (Agilent, Santa Clara, CA, USA).

### 3.2. Experimental Part

*1-((2R,3S)-3-Ethyl-2-vinylpiperidin-1-yl)ethan-1-one* (**17**). To a cooled (0 °C) solution of piperidine **18** (1.0 g, 5.0 mmol) in CH_2_Cl_2_ (15 mL) were added DMAP (catalytic amount), Et_3_N (2.0 mL, 15.0 mmol), and acetyl chloride (0.5 mL, 7.5 mmol). The mixture was stirred for 2 h at room temperature, quenched with water, and extracted with CH_2_Cl_2_. The combined organic layer was washed with brine, dried over MgSO_4_, and concentrated *in vacuo*. The residue was purified uisng flash column chromatography (EtOAc/Hexane = 1:2) to provide 770 mg (85%) of **17**. [α]${}_{D}^{20}$ = −33.66 (*c* 1.08, CHCl_3_); ^1^H-NMR (CDCl_3_, 500 MHz, mixture of rotamers) δ 5.82–5.73 (m, 1H), 5.23–5.19 (m, 1.5H), 5.07–5.01 (m, 1H), 4.46 (d, *J* = 11.3 Hz, 0.5H), 4.18 (s, 0.5H), 3.54 (d, *J* = 13.1 Hz, 0.5H), 3.17 (t, *J* = 11.8 Hz, 0.5H), 2.64 (t, *J* = 12.8 Hz, 0.5H), 2.11 (s, 1.5H), 2.05 (s, 1.5H), 1.64 (m, 3H), 1.50–1.43 (m, 3H), 1.40–1.28 (m, 1H), 0.92 (t, *J* = 6.6 Hz, 3H); ^13^C-NMR (CDCl_3_, 100 MHz, mixture of rotamers) δ 170.7, 137.0, 136.7, 116.1, 115.9, 115.8, 59.6, 53.6, 53.5, 42.4, 42.3, 39.7, 38.7, 37.0, 24.1, 23.4, 23.3, 23.1, 21.3, 20.8, 19.6, 12.2; IR (thin film, neat) *ν*_max_ 3477, 2937, 1651, 1423, 1267 cm^−1^; HR-MS (ESI+) calcd. for C_11_H_19_NNaO [M + Na]^+^ 204.1359; found 204.1360.

*(S,E)-7-Ethyl-3,4,7,8,9,10-hexahydroazecin-2(1H)-one* (**19**). To a refluxing solution of amide **17** (750 mg, 4.1 mmol) in toluene (40 mL) was added lithium bis(trimethylsilyl)amide (LiHMDS) (1.0 M in toluene, 12.4 mL, 12.4 mmol). The mixture was stirred for 1 h at the same temperature, cooled to room temperature, quenched with water, and extracted with CH_2_Cl_2_. The combined organic layer was washed with brine, dried over MgSO_4_, and concentrated *in vacuo*. The residue was purified using flash column chromatography (EtOAc/Hexane = 1:1) to provide 540 mg (72%) of **19**. [α]${}_{D}^{20}$ = +15.07 (*c* 0.55, CHCl_3_); ^1^H-NMR (CDCl_3_, 600 MHz) δ 5.76 (br s, 1H), 5.27 (ddd, *J* = 15.1, 10.1, 5.0 Hz, 1H), 5.00 (dd, *J* = 15.6, 9.6 Hz, 1H), 3.39 (dd, *J* = 13.8, 7.5 Hz, 1H), 2.76 (dd, *J* = 12.8, 8.2 Hz, 1H), 2.22–2.11 (m, 3H), 1.95 (td, *J* = 11.3, 5.5 Hz, 1H), 1.74–1.70 (quint, *J* = 6.6 Hz, 2H), 1.59–1.57 (m, 1H), 1.25–1.14 (m, 3H), 1.13–1.06 (m, 1H), 0.69 (t, *J* = 7.4 Hz, 3H); ^13^C-NMR (CDCl_3_, 150 MHz) δ 173.0, 138.7, 126.6, 45.8, 40.5, 38.5, 35.7, 29.5, 28.2, 26.7, 11.8; IR (thin film, neat) *ν*_max_ 3309, 2926, 1645, 1554 cm^−1^; HR-MS (ESI+) calcd. for C_11_H_20_NO [M + H]^+^ 182.1539; found 182.1532.

*tert-Butyl (S,E)-5-ethyl-10-oxo-3,4,5,8,9,10-hexahydroazecine-1(2H)-carboxylate* (**16**). To a cooled (−78 °C) solution of lactam **19** (530 mg, 2.9 mmol) in THF (9 mL) was slowly added *n*BuLi (2.5 M in hexane, 1.8 mL, 4.5 mmol). The mixture was stirred for 10 min at the same temperature and a solution of Boc_2_O (2.8 mL, 6.1 mmol) in THF (3 mL) was added. The reaction mixture was stirred for 1 h 50 min at the same temperature, quenched with water, and extracted with EtOAc. The combined organic layer was washed with brine, dried over MgSO_4_, and concentrated *in vacuo*. The residue was purified using flash column chromatography (EtOAc/Hexane = 1:20) to provide 814 mg (99%) of **16**. [α]${}_{D}^{20}$ = −16.08 (*c* 0.98, CHCl_3_); ^1^H-NMR (CDCl_3_, 300 MHz) δ 5.36–5.26 (m, 1H), 5.01(br s, 1H), 3.70–3.56 (m, 2H), 3.21 (br s, 1H), 2.80 (br s, 1H), 2.80–2.31 (m, 2H), 1.71–1.67 (m, 2H), 1.56 (s, 1H), 1.53 (s, 10H), 1.34–1.22 (m, 3H), 0.80 (t, *J* = 8.9 Hz, 3H); ^13^C-NMR (CDCl_3_, 125 MHz, mixture of rotamers) δ 177.5, 153.5, 146.7, 138.9, 126.0, 85.1, 82.5, 47.1, 46.3, 39.3, 33.4, 33.1, 30.9, 29.2, 28.9, 28.1, 27.8, 27.6, 27.4, 24.8, 24.7 12.1; IR (thin film, neat) *ν*_max_ 2961, 1726, 1691, 1369, 1148 cm^−1^; HR-MS (ESI+) calcd. for C_16_H_27_NNaO_3_ [M + Na]^+^ 304.1883; found 304.1867.

*tert-Butyl (5S,10S,E)-5-ethyl-10-(2-methoxy-2-oxoethyl)-3,4,5,8,9,10-hexahydroazecine-1(2H)-carboxylate* (**15**). To a cooled (−78 °C) solution of lactam **16** (860 mg, 3.1 mmol) in CH_2_Cl_2_ (9 mL) was slowly added diisobutylaluminium hydride (DIBAL-H) (1.0 M in toluene, 5.5 mL, 5.5 mmol). After stirring for 10 min at the same temperature, pyridine (1.2 mL, 15.3 mmol) and trimethylsilyl trifluoromethanesulfonate (TMSOTf) (1.4 mL, 7.7 mmol) were added. The reaction mixture was stirred for 10 min at the same temperature, quenched with saturated Rochelle’s solution, and extracted with Et_2_O. The combined organic layer was washed with brine, dried over MgSO_4_, and concentrated *in vacuo*. The residue was purified using flash column chromatography (EtOAc/Hexane = 1:20, silica gel deactivated with Et_3_N) to afford unstable *N*,*O*-acetal TMS ether **20**. To a cooled (−78 °C) solution of **20** in CH_2_Cl_2_ (9 mL) were added 1-(*tert*-butyldimethylsilyloxyl)-1-methoxyethane (1.1 mL, 4.9 mmol) **21** and BF_3_∙OEt_2_ (0.3 mL. 2.7 mmol). After stirring for 30 min at the same temperature, the reaction mixture was allowed to warm to 0 °C. The reaction mixture was quenched with Et_3_N and concentrated *in vacuo*. The residue was purified using flash column chromatography (EtOAc/Hexane = 1:10) to provide 783 mg of **15** as a diastereomeric mixture (75% for 2 steps, 68% for desired diastereomer). [α]${}_{D}^{20}$ = +37.66 (*c* 2.36, CHCl_3_); ^1^H-NMR (CDCl_3_, 500 MHz, mixture of rotamers) δ 5.51–5.42 (m, 1H), 5.20–5.13 (m, 1H), 3.71 (m, 0.5H), 3.61 (s, 3H), 3.20 (br s, 0.5H), 3.11–3.10 (m, 1H), 2.82 (dd, *J* = 15.0, 8.8 Hz, 0.5H), 2.53–2.47 (m, 1H), 2.43–2.37 (m, 0.5H), 2.31–2.26 (m, 1.5H), 2.19 (br s, 0.5H), 2.08 (br s, 0.5H), 1.98–1.89 (m, 1.5H), 1.83–1.76 (m, 1H), 1.67–1.62 (m, 1H), 1.45–1.39 (m, 10.5H), 1.36–1.29 (m, 1.5H), 1.27–1.15 (m, 2H), 0.93 (t, *J* = 12.2 Hz, 1H), 0.82 (t, *J* = 7.4 Hz, 3H); ^13^C-NMR (CDCl_3_, 150 MHz, mixture of rotamers) δ 173.0, 172.4, 155.3, 155.0, 135.2, 134.6, 131.5, 130.9, 79.6, 78.9, 64.4, 56.0, 51.6, 51.4, 48.3, 48.1, 40.3, 38.5, 35.9, 35.8, 34.4, 34.1, 33.5, 31.6, 30.6, 29.2, 28.6, 28.5, 28.4, 25.6, 22.6, 14.0, 12.2; IR (thin film, neat) *ν*_max_ 2961, 2930, 1740, 1692, 1365, 1172 cm^−1^; LR-MS (FAB+) *m*/*z* 340 [M + H]^+^; HR-MS (FAB+) calcd. for C_19_H_34_NO_4_ [M + H]^+^ 340.2488; found 340.2484.

*tert-Butyl (2S,7R)-7-ethyl-2-(2-methoxy-2-oxoethyl)azecane-1-carboxylate* (**23**). To a solution of ester **15** (705 mg, 2.1 mmol) in MeOH (10 mL) was added 10% Pd/C (71 mg) and the mixturre was stirred under H_2_ (balloon pressure) for 19 h. The reaction mixture was filtered through Celite^®^ and concentrated *in vacuo*. The residue was purified using flash column chromatography (EtOAc/Hexane = 1:10) to provide 688 mg (97%) of **23**. [α]${}_{D}^{20}$ = +23.72 (*c* 2.29, CHCl_3_); ^1^H-NMR (CDCl_3_, 500 MHz) δ 3.75 (br s, 1H), 3.61 (s, 3H), 3.49 (br s, 1H), 2.99–2.65 (m, 2H), 2.44–2.40 (m, 1H), 2.06–1.91 (m, 1H), 1.67 (br s, 2H), 1.41 (br s, 15H), 1.30 (s, 2H), 1.25–1.20 (m, 2H), 1.09 (br s, 2H), 0.81 (t, *J* = 7.2 Hz, 3H); ^13^C-NMR (CDCl_3_, 125 MHz) δ 172.5, 155.9, 79.7, 79.2, 56.1, 51.6, 39.1, 38.3, 37.0, 32.8, 30.8, 30.1, 28.5, 27.8, 26.7, 22.7, 11.9; IR (thin film, neat) *ν*_max_ 2959, 2925, 1741, 1696, 1365, 1171 cm^−1^; LR-MS (FAB+) *m*/*z* 342 [M + H]^+^; HR-MS (FAB+) calcd. for C_19_H_36_NO_4_ [M + H]^+^ 342.2644; found 342.2644.

*tert-Butyl (2S,7R)-2-((E)-2-((tert-butyldimethylsilyl)oxy)vinyl)-7-ethylazecane-1-carboxylate* (**24**). To a cooled (−78 °C) solution of ester **23** (645 mg, 1.9 mmol) in CH_2_Cl_2_ (8 mL) was slowly added DIBAL-H (1.0 M in toluene, 2.4 mL, 2.4 mmol). The reaction mixture was stirred for 30 min at the same temperature, quenched with saturated Rochelle’s solution, and extracted with CH_2_Cl_2_. The combined organic layer was washed with brine, dried over MgSO_4_, and concentrated *in vacuo*. The crude aldehyde was directly used for the next reaction without further purification. To a stirred solution of aldehyde in CH_2_Cl_2_ (9 mL) were added *tert*-butyldimethylsilyl chloride (TBSCl) (570 mg, 3.8 mmol) and DBU (0.9 mL, 6.0 mmol). The reaction mixture was refluxed for 1 h and concentrated *in vacuo*. The residue was purified using flash column chromatography (EtOAc/Hexane = 1:30, silica gel deactivated with Et_3_N) to provide 563 mg (70% for 2 steps) of **24**. [α]${}_{D}^{20}$ = +26.68 (*c* 1.25, CHCl_3_); ^1^H-NMR (CDCl_3_, 500 MHz) δ 6.31 (dd, *J* = 15.8, 9.4 Hz, 1H), 5.10 (m, 1H), 3.64 (m, 1H), 2.85–2.78 (m, 1H), 2.04 (m, 1H), 1.74 (br s, 1H), 1.66–1.63 (m, 1H), 1.54 (s, 1H), 1.44 (s, 11H), 1.35 (m, 3H), 1.25–1.18 (m, 4H), 1.14 (br s, 2H), 0.89 (s, 9H), 0.86–0.84 (m, 4H), 0.11 (s, 6H); ^13^C-NMR (CDCl_3_, 125 MHz) δ 156.3, 141.3, 138.6, 111.9, 79.2, 78.8, 57.2, 36.4, 32.6, 30.0, 28.5, 26.2, 25.7, 25.6, 22.8, 18.4, 18.1, 11.9, −3.0, −4.8; IR (thin film, neat) *ν*_max_ 2958, 2929, 1695, 1655, 1170, 839 cm^−1^; LR-MS (FAB+) *m*/*z* 426 [M + H]^+^; HR-MS (FAB+) calcd. for C_24_H_48_NO_3_Si [M + H]^+^ 426.3403; found 426.3394.

*1-((2S,7R)-2-((E)-2-((tert-Butyldimethylsilyl)oxy)vinyl)-7-ethylazecan-1-yl)propan-1-one* (**14**). To a cooled (0 °C) solution of silyl enol ether **24** (69.4 mg, 0.16 mmol) in CH_2_Cl_2_ (2 mL) were added 2,6-lutidine (0.1 mL, 0.64 mmol) and TMSOTf (0.1 mL, 0.48 mmol). The mixture was stirred for 30 min at the same temperature, quenched with MeOH, and concentrated *in vacuo*. The crude amine **25** was directly used for the next reaction without further purification. To a stirred solution of amine **25** in CH_2_Cl_2_ (2 mL) were added DMAP (catalytic amount), Et_3_N (0.1 mL, 0.48 mmol), and propionic anhydride (0.04 mL, 0.32 mmol). The reaction mixture was stirred for 1 h and concentrated *in vacuo*. The residue was purified using flash column chromatography (EtOAc/Hexane = 1:10, silica gel deactivated with Et_3_N) to provide 49.8 mg (80% for 2 steps) of **14**. [α]${}_{D}^{20}$ = +12.16 (*c* 2.00, CHCl_3_); ^1^H-NMR (CDCl_3_, 500 MHz) δ 6.31 (d, *J* = 15.5 Hz, 1H), 6.22 (d, *J* = 15.5 Hz, 1H), 5.23 (br s, 1H), 4.93 (dd, *J* = 12.1, 6.6 Hz, 1H), 4.13 (t, *J* = 8.6 Hz, 1H), 3.49–3.37 (m, 2H), 3.08–2.92 (m, 2H), 2.38–2.27 (m, 2H), 2.24 (qd, *J* = 7.5, 2.4 Hz, 3H), 2.03 (m, 1H), 1.89–1.86 (m, 1H), 1.75 (br s, 1H), 1.65–1.55 (m, 2H), 1.49–1.47 (m, 1H), 1.39 (m, 1H), 1.26–1.13(m, 2H), 1.11–1.06 (m, 2H), 0.85 (s, 9H), 0.82 (t, *J* = 7.4 Hz, 3H), 0.07 (s, 6H); ^13^C-NMR (CDCl_3_, 125 MHz, mixture of rotamers) δ 175.0, 174.8, 174.5, 142.6, 142.0, 141.7, 111.8, 111.7, 111.6, 56.6, 56.1, 41.8, 39.4, 37.4, 36.7, 32.7, 31.6, 31.0, 31.0, 30.8, 30.7, 30.5, 29.8, 29.7, 29.7, 29.5, 29.4, 29.4, 29.3, 29.2, 28.2, 28.0, 27.8, 27.5, 27.4, 25.7, 25.6, 25.5, 25.5, 25.2, 25.0, 24.8, 24.5, 24.2, 23.8, 23.3, 18.3, 11.9, 9.4, −5.3; IR (thin film, neat) *ν*_max_ 2956, 2930, 1653, 839 cm^−1^; LR-MS (FAB+) *m*/*z* 382 [M + H]^+^; HR-MS (FAB+) calcd. for C_22_H_44_NO_2_Si [M + H]^+^ 382.3141; found 382.3139.

*(3S,4R,11R,E)-4-((tert-Butyldimethylsilyl)oxy)-11-ethyl-3-methylazacyclotetradec-5-en-2-one* (**26**). To a heated (60 °C) solution of amide **14** (46.6 mg, 0.13 mmol) in benzene (3 mL) was slowly added *i*PrMgCl (1.0 M in hexane, 0.5 mL, 0.5 mmol). The reaction mixture was stirred for 40 min at the same temperature, cooled to room temperature, quenched with water, and extracted with EtOAc. The combined organic layer was washed with brine, dried over MgSO_4_, and concentrated *in vacuo*. The residue was purified using flash column chromatography (EtOAc/Hexane = 1:10) to provide 46.1 mg of **26** as a diastereomeric mixture (99%, 95% for desired diastereomer). [α]${}_{D}^{20}$ = −62.95 (*c* 0.62, CHCl_3_); ^1^H-NMR (CDCl_3_, 500 MHz) δ 6.25 (s, 1H), 5.59–5.54 (m, 1H), 5.37 (dd, *J* = 15.5, 5.8 Hz, 1H), 4.18 (t, *J* = 6.5 Hz, 1H), 3.89–3.83 (m, 1H), 2.51 (m, 1H), 2.27 (quint, *J* = 7.1 Hz, 1H), 2.01 (m, 2H), 1.56 (s, 1H), 1.48–1.38 (m, 2H), 1.36–1.20 (m, 8H), 1.17 (d, *J* = 7.0 Hz, 3H), 1.07–0.99 (m, 2H), 0.89 (s, 9H), 0.83 (t, *J* = 7.3 Hz, 3H), 0.06 (s, 3H), 0.02 (s,3H); ^13^C-NMR (CDCl_3_, 125 MHz) δ 173.9, 131.8, 130.7, 75.0, 48.4, 39.4, 37.7, 31.0, 30.9, 27.2, 27.1, 26.4, 25.9, 23.8, 23.6, 18.1, 15.1, 12.0, −4.3, −4.9; IR (thin film, neat) *ν*_max_ 3279, 2928, 1638, 775 cm^−1^; LR-MS (FAB+) *m*/*z* 382 [M + H]^+^; HR-MS (FAB+) calcd. for C_22_H_44_NO_2_Si [M + H]^+^ 382.3141; found 382.3140.

*Crystal Data for***26**. C_22_H_43_NO_2_Si (M = 381.67 g/mol), orthorhombic, space group P2_1_2_1_2 (no. 18), a = 9.4913(8) Å, b = 31.653(3) Å, c = 8.524(1) Å, V = 2560.9(5) Å^3^, Z = 4, T = 300 K, µ(MoKa) = 1.052 mm^−1^, D_calc_ = 0.990 g/cm^3^, 25442 reflections measured, 5869 unique (R_int_ = 0.1257) which were used in all calculations. The final R1 was 0.1071 (I > 2σ(I)) and wR2 was 0.2824 (all data). 

*(−)-6-Desmethyl*-*fluvirucinine A_1_* (**13**). To a solution of lactam **26** (39.8 mg, 0.10 mmol) in a mixture of EtOAc and MeOH (1:1, 2 mL) was added 10% Pd/C (4.0 mg) and the mixture was stirred under H_2_ (balloon pressure) for 12 h. The reaction mixture was filtered through Celite^®^ and concentrated *in vacuo*. The crude lactam **27** was directly used for the next reaction without further purification. To a stirred solution of lactam **27** in THF (1 mL) was added tetrabutylammonium fluoride (TBAF) (1.0 M in THF, 0.2 mL, 0.20 mmol) at room temperature. The mixture was stirred for 1 h at the same temperature, quenched with water, and extracted with EtOAc. The combined organic layer was washed with brine, dried over MgSO_4_, and concentrated *in vacuo*. The residue was purified by flash column chromatography (EtOAc/Hexane/MeOH = 25:25:1) to provide 27.8 mg (99% for 2 steps) of **13**. [α]${}_{D}^{20}$ = −76.61 (*c* 0.18, MeOH); ^1^H-NMR (MeOD, 500 MHz) δ 4.58 (s, 1H), 3.68–3.62 (m, 2H), 2.69 (ddd, *J* = 13.6, 7.7, 1.7 Hz, 1H), 2.37–2.31(m, 1H), 1.62–1.52 (m, 2H), 1.50–1.45 (m, 4H), 1.43–1.41 (m, 4H), 1.39–1.32 (m, 3H), 1.30–1.26 (m, 2H), 1.24–1.10 (m, 4H), 1.17 (d, *J* = 6.9 Hz, 3H), 0.86 (t, *J* = 7.4 Hz, 3H); ^13^C-NMR (MeOD, 150 MHz) δ 178.7, 74.6, 48.8, 40.3, 38.5, 35.3, 32.6, 29.8, 28.9, 28.7, 27.8, 27.5, 24.2, 22.7, 16.8, 12.7; IR (thin film, neat) *ν*_max_ 3388, 3305, 1637, 790 cm^−1^; HR-MS (ESI+) calcd. for C_16_H_31_NO_2_ [M + H]^+^ 270.2428; found 270.2426.

## 4. Conclusions

The versatile synthesis of (−)-6-desmethyl-fluvirucinine A_1_ (**13**) was accomplished through 13 steps with a 24% overall yield from the known vinylpiperidine **18**. The key part of the synthesis included the highly diastereoselective ACR of the 10-membered lactam intermediate for the elaboration of the 14-membered lactam framework via the conformationally-induced diastereocontrol of the distal stereocenters. The stereochemical effect of 6-desmethyl-fluvirucinine A_1_ on the biological activities and the synthesis of the carbohydrate moiety will be reported in future research.

## Supplementary Materials

The supplementary materials are available online.

## References

[1] Naruse N., Tenmyo O., Kawano K., Tomita K., Ohgusa N., Miyaki T., Konishi M., Oki T. (1991). Fluvirucins A_1_, A_2_, B_1_, B_2_, B_3_, B_4_ and B_5_, new antibiotics active against influenza A virus. I. Production, isolation, chemical properties and biological activities. J. Antibiot..

[2] Naruse N., Tsuno T., Sawada Y., Konishi M., Oki T. (1991). Fluvirucins A_1_, A_2_, B_1_, B_2_, B_3_, B_4_ and B_5_, new antibiotics active against influenza A virus. II. Structure determination. J. Antibiot..

[3] Naruse N., Konishi M., Oki T., Inouye Y., Kakisawa H. (1991). Fluvirucins A_1_, A_2_, B_1_, B_2_, B_3_, B_4_ and B_5_, new antibiotics active against influenza A virus. III. The stereochemistry and absolute configuration of fluvirucin A_1_. J. Antibiot..

[4] Tomita K., Oda N., Hoshino Y., Ohkusa N., Chikazawa H. (1991). Fluvirucins A_1_, A_2_, B_1_, B_2_, B_3_, B_4_ and B_5_, new antibiotics active against influenza A virus. IV. Taxonomy on the producing organisms. J. Antibiot..

[5] Hegde V.R., Patel M.G., Gullo V.P., Ganguly A.K., Sarre O., Puar M.S., McPhail A.T. (1990). Macrolactams: A new class of antifungal agents. J. Am. Chem. Soc..

[6] Hegde V., Patel M., Horan A., Gullo V., Marquez J., Gunnarsson I., Gentile F., Loebenberg D., King A., Puar M. (1992). Macrolactams: A novel class of antifungal antibiotics produced by *Actinomadura* spp. SCC 1776 and SCC 1777. J. Antibiot..

[7] Houri A.F., Xu Z.M., Cogan D.A., Hoveyda A.H. (1995). Cascade catalysis in synthesis. an enantioselective route to Sch-38516 (and fluvirucin B_1_) aglycon macrolactam. J. Am. Chem. Soc..

[8] Xu Z.M., Johannes C.W., Salman S.S., Hoveyda A.H. (1996). Enantioselective total synthesis of antifungal agent Sch 38516. J. Am. Chem. Soc..

[9] Xu Z.M., Johannes C.W., Houri A.F., La D.S., Cogan D.A., Hofilena G.E., Hoveyda A.H. (1997). Applications of Zr-catalyzed carbomagnesation and Mo-catalyzed macrocyclic ring closing metathesis in asymmetric synthesis. Enantioselective total synthesis of Sch 38516 (Fluvirucin B_1_). J. Am. Chem. Soc..

[10] Trost B.M., Ceschi M.A., Konig B. (1997). Palladium-catalyzed additions of alkenyl epoxides to pronucleophiles: A synthesis of the macrolactam aglycone of fluviricin B_1_. Angew. Chem. Int. Ed..

[11] Martin M., Mas G., Urpi F., Vilarrasa J. (1999). High-yielding enantioselective synthesis of the macrolactam aglycon of Sch 38516 from two units of (2*R*)-2-ethyl-4-penten-1-ol. Angew. Chem. Int. Ed..

[12] Liang B., Negishi E. (2008). Highly efficient asymmetric synthesis of fluvirucinine A_1_, via Zr-catalyzed asymmetric carboalumination of alkenes (ZACA)-lipase-catalyzed acetylation tandem process. Org. Lett..

[13] Llacer E., Urpi F., Vilarrasa J. (2009). Efficient approach to fluvirucins B_2_, B_5_, Sch 38518, and Sch 39185. First synthesis of their aglycon, via CM and RCM reactions. Org. Lett..

[14] Suh Y.-G., Kim S.-A., Jung J.-K., Shin D.-Y., Min K.-H., Koo B.-A., Kim H.-S. (1999). Asymmetric total synthesis of fluvirucinine A_1_. Angew. Chem. Int. Ed..

[15] Lee Y.-S., Jung J.-W., Kim S.-H., Jung J.-K., Paek S.-M., Kim N.-J., Chang D.-J., Lee J., Suh Y.-G. (2010). First total synthesis and structural confirmation of fluvirucinine A_2_ via an iterative ring expansion strategy. Org. Lett..

[16] Ayers S., Zink D.L., Mohn K., Powell J.S., Brown C.M., Murphy T., Grund A., Genilloud O., Salazar O., Thompson D. (2007). Anthelmintic macrolactams from *Nonomuraea turkmeniaca* MA7364. J. Nat. Prod..

[17] Ayers S., Zink D.L., Powell J.S., Brown C.M., Grund A., Genilloud O., Salazar O., Thompson D., Singh S.B. (2008). Anthelmintic macrolactams from *Nonomuraea turkmeniaca* MA7381. J. Antibiot..

[18] Majumdar K.C., Bhattacharyya T., Chattopadhyay B., Sinha B. (2009). Recent advances in the aza-Claisen rearrangement. Synthesis.

[19] Jung J.-W., Kim S.-H., Suh Y.-G. (2017). Advances in aza-Claisen-rearrangement-induced ring-expansion strategies. Asian J. Org. Chem..

[20] Castro A.M.M. (2004). Claisen rearrangement over the past nine decades. Chem. Rev..

[21] Suh Y.-G., Lee J.-Y., Kim S.-A., Jung J.-K. (1996). A new ring expansion reaction of 1-acyl-2-vinylpiperidine and 1-acyl-2-vinylpiperazine via aza-Claisen rearrangement of amide enolate. Synth. Commun..

[22] Jung J.-K., Choi N.-S., Suh Y.-G. (2004). Functional divergency oriented synthesis of azoninones as the key intermediates for bioactive indolizidine alkaloids analogs. Arch. Pharm. Res..

[23] Suh Y.-G., Lee Y.-S., Kim S.-H., Jung J.-K., Yun H., Jang J., Kim N.-J., Jung J.-W. (2012). A stereo-controlled access to functionalized macrolactams via an aza-Claisen rearrangement. Org. Biomol. Chem..

[24] Yun H., Kim J., Sim J., Lee S., Han Y.T., Chang D.-J., Kim D.-D., Suh Y.-G. (2012). Asymmetric syntheses of 1-deoxy-6,8a-di-*epi*-castanospermine and 1-deoxy-6-*epi*-castanospermine. J. Org. Chem..

[25] Paek S.-M., Kim N.-J., Shin D., Jung J.-K., Jung J.-W., Chang D.-J., Moon H., Suh Y.-G. (2010). A concise total synthesis of (+)-tetrabenazine and (+)-α-dihydrotetrabenazine. Chem. Eur. J..

[26] Jang J., Jung J.-W., Ahn J., Sim J., Chang D.-J., Kim D.-D., Suh Y.-G. (2012). Asymmetric formal synthesis of schulzeines A and C. Org. Biomol. Chem..

[27] Sim J., Yun H., Jung J.-W., Lee S., Kim N.-J., Suh Y.-G. (2012). Achiral auxiliary-assisted chiral transfer via microwave-accelerated aza-Claisen rearrangement: A short synthesis of (+)-1-hydroxyquinolizidinone. Tetrahedron Lett..

[28] Moon H., An H., Sim J., Kim K., Paek S.-M., Suh Y.-G. (2015). Efficient strategy for the stereoselective synthesis of 2,3-disubstituted benzo[α]quinolizidine alkaloids: Concise synthesis of (−)-protoemetinol. Tetrahedron Lett..

[29] Suh Y.-G., Lim C., Sim J., Lee J.K., Surh Y.-J., Paek S.-M. (2017). Construction of the azacyclic core of tabernaemontanine-related alkaloids via tandem Reformatsky aza-Claisen rearrangement. J. Org. Chem..

[30] Suh Y.-G., Shin D.-Y., Jung J.-K., Kim S.-H. (2002). The versatile conversion of acyclic amides to α-alkylated amines. Chem. Commun..

[31] Suh Y.-G., Kim S.-H., Jung J.-K., Shin D.-Y. (2002). The versatile conversion of lactams to the α-alkylated azacycles via cyclic *N*,*O*-acetal TMS ether. Tetrahedron Lett..

[32] Jung J.-W., Shin D.-Y., Seo S.-Y., Kim S.-H., Paek S.-M., Jung J.-K., Suh Y.-G. (2005). A new entry to functionalized cycloalkylamines: Diastereoselective intramolecular amidoalkylation of *N*,*O*-acetal TMS ether possessing allylsilane. Tetrahedron Lett..

[33] Suh Y.-G., Jang J., Yun H., Han S.M., Shin D., Jung J.-K., Jung J.-W. (2011). Expedient synthesis of chiral homoallylamines via *N*,*O*-acetal TMS ethers and its application. Org. Lett..

[34] Shin D.-Y., Jung J.-K., Seo S.-Y., Lee Y.-S., Paek S.-M., Chung Y.K., Shin D.M., Suh Y.-G. (2003). Stereoselective formation of *N*-acyliminium ion via chiral *N*,*O*-acetal TMS ether and its application to the synthesis of β-amino acids. Org. Lett..

[B35-molecules-23-02351] 35.Geometry optimization of (*Z*)-*N*-acyl iminium intermediate **22** was performed using ChemBio3D (Ver 14.0), Avogadro, and MOPAC. Details of the geometry opimization are provided in the Supplementary Materials.

[36] Taniguchi Y., Inanaga J., Yamaguchi M. (1981). Use of 1,8-diazabicyclo[5.4.0]undec-7-ene in preparation of trimethylsilyl enol ethers and trimethylsilylacetylenes. Bull. Chem. Soc. Jpn..

[37] Fleming I., Barbero A., Walter D. (1997). Stereochemical control in organic synthesis using silicon-containing compounds. Chem. Rev..

[38] Cambridge Crystallographic Data Centre; CCDC 1864701 (Online); 12 Union Road, Cambridge CB2 1EZ, UK. http://www.ccdc.cam.ac.uk/conts/retrieving.html.

