# Supplementary material for: Asymmetric Synthesis of (−)-6-Desmethyl-Fluvirucinine A1 via Conformationally-Controlled Diastereoselective Lactam-Ring Expansions
Source: Molecules. 2018 Sep 14;23(9):2351. doi: 10.3390/molecules23092351 (PMC6225218; doi:10.3390/molecules23092351)
Supplement: Supplementary file 1 [file molecules-23-02351-s001.pdf]

Asymmetric Synthesis of (-)-6-Desmethyl-Fluvirucinine A_1 via Conformationally-Controlled Diastereoselective Lactam-Ring Expansions

Hyunyoung Moon[1,2,†], Hojong Yoon[2,†], Changjin Lim[1,2], Jaebong Jang[2], Jong-Jae Yi[1], Jae Kyun Lee[3], Jeeyeon Lee[2], Younghwa Na[1], Woo Sung Son[1], Seok-Ho Kim[1], and Young-Ger Suh[1,2,*]

[1]*College of Pharmacy, Cha University, 120 Haeryong-ro, Pocheon 11160, Gyeonggi-do, Korea; hyunyoungmoon@gmail.com (H.M); koryoi0709@gmail.com (C.L); nmryi222@gmail.com (J.-J.Y); yna7315@cha.ac.kr (Y.N); wson@cha.ac.kr (W.S.S)*

[2]*College of Pharmacy, Seoul National University, 1 Gwanak-ro, Gwanak-gu, Seoul 08826, Korea; hojong_yoon@g.harvard.edu (H.Y); jaebong.jang@gmail.com (J.J); jyleeut@snu.ac.kr (J.L)*

[3]*Center for Neuro-Medicine, Korea Institute of Science and Technology (KIST), Seoul, 02792; j9601@kist.re.kr (J.K.L)*

Correspondence: ygsuh@snu.ac.kr (or ygsuh@cha.ac.kr) (Y.-G.S) Tel.: +82-31-850-9300; ksh@cha.ac.kr (S.-H.K) Tel.: +82-31-881-7169.

[†]*These authors contributed equally to this work.*

Supporting Information

List of Contents

I. ^{1}H NMR and ^{13}C NMR spectra of **13** ········ S2

II. Geometry optimization ········ S3

III. X-ray crystallographic data ········ S4

I. ^{1}H NMR and ^{13}C NMR spectra of **13**

^{1}H NMR (MeOD, 500 MHz) of **13**

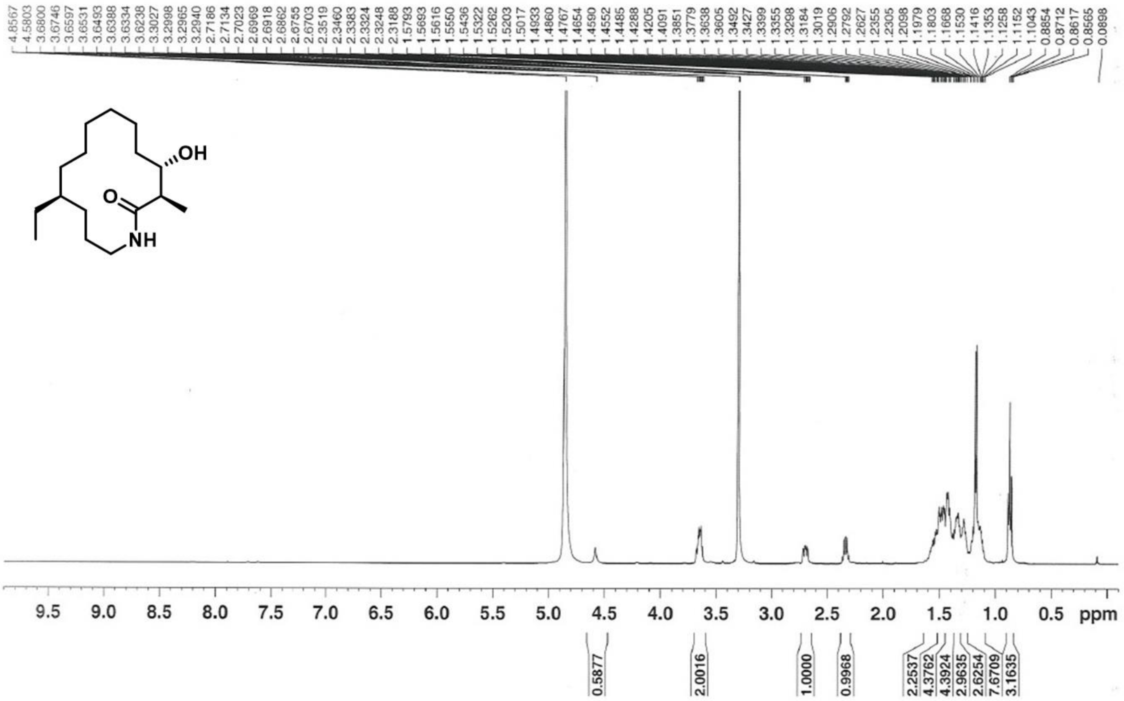

^{13}C NMR (MeOD, 125 MHz) of **13**

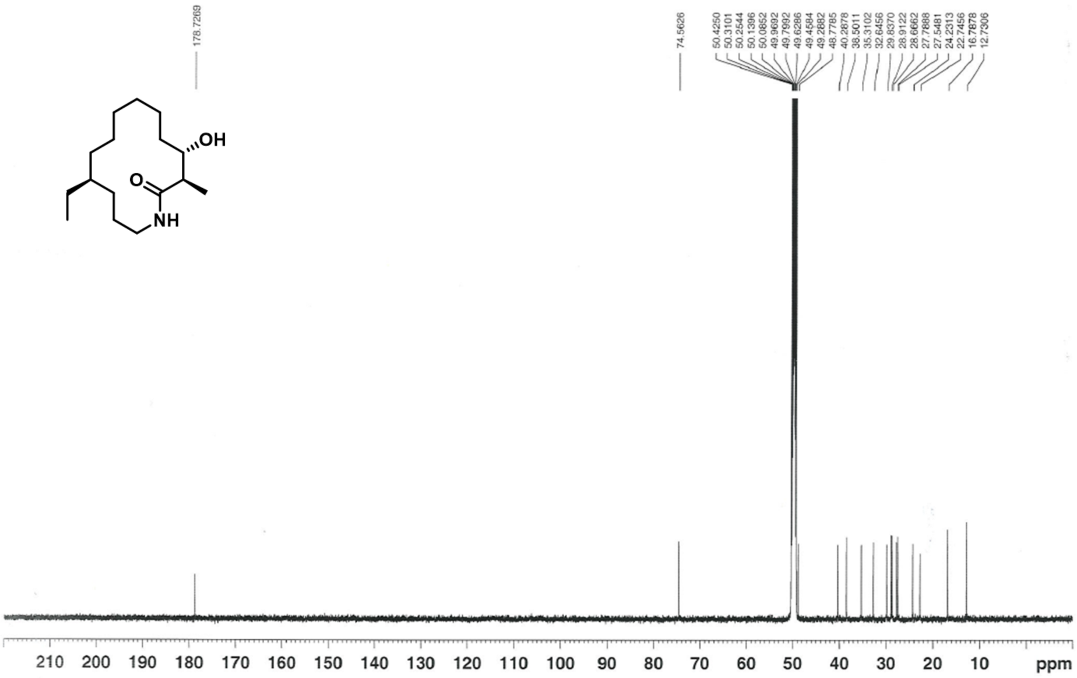

II. Geometry optimization

Geometry optimization of (*Z*)-*N*-acyl iminium intermediate **22** was performed using ChemBio3D (Ver 14.0)[1], Avogadro[2], and MOPAC[3]. Initial conformation of (*Z*)-*N*-acyl iminium intermediate **22** was obtained by molecular mechanics minimization with MM2 force field. Further minimization of resulting structures was performed in UFF, and MMF94 force field iteratively until values of energy reached the minimum. The final minimized values of energy were calculated using Avogadro and MOPAC. All structures were represented with UCSF Chimera[4], and Avogadro.

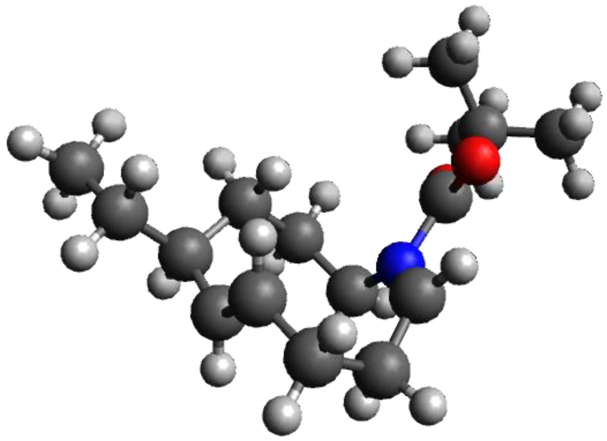

Total energy : 14.7661 kJ/mol

References

1. ChemBioOffice Ultra 14, CambridgeSoft, **2014**.

2. Marcus D Hanwell, Donald E Curtis, David C Lonie, Tim Vandermeersch, Eva Zurek and Geoffrey R Hutchison, Avogadro: An advanced semantic chemical editor, visualization, and analysis platform, *J. Cheminformatics* **2012**, *4*,17.

3. James J. P. Stewart, Stewart, MOPAC2016, Computational Chemistry, Colorado Springs, CO, USA, HTTP://OpenMOPAC.net, **2016**.

4. Pettersen EF, Goddard TD, Huang CC, Couch GS, Greenblatt DM, Meng EC, Ferrin TE, UCSF Chimera--a visualization system for exploratory research and analysis, *J. Comput. Chem.* **2004**, *25*, 1605.

III. X-ray crystallographic data

Empirical Formula	$C_{22}H_{43}NO_2Si$
Formula Weight	381.67
Crystal Color, Habit	colorless, block
Crystal Dimensions	0.300 X 0.200 X 0.200 mm
Crystal System	orthorhombic
Lattice Type	Primitive
Lattice Parameters	a = 9.4913(8) Å<br>b = 31.653(3) Å<br>c = 8.524(1) Å<br>V = 2560.9(5) Å^3
Space Group	$P2_12_12$ (#18)
Z value	4
D_{calc}	0.990 g/cm^3
F_{000}	848.00
μ(MoKα)	1.052 cm^{-1}

B. Intensity Measurements

Diffractometer	R-AXIS RAPID
Radiation	MoKα (λ = 0.71075 Å)<br>graphite monochromated
Voltage, Current	50kV, 30mA
Temperature	23.0^{o}C
Detector Aperture	280 x 256 mm
Data Images	44 exposures
ω oscillation Range (χ=45.0, φ=0.0)	130.0 - 190.0^{o}
Exposure Rate	60.0 sec./^{o}
ω oscillation Range (χ=45.0, φ=180.0)	0.0 - 160.0^{o}
Exposure Rate	60.0 sec./^{o}
Detector Position	127.40 mm
Pixel Size	0.100 mm
$2\theta_{max}$	55.0^{o}
No. of Reflections Measured	Total: 25442<br>Unique: 5869 (R_{int} = 0.1257)<br>Friedel pairs: 2518
Corrections	Lorentz-polarization<br>Absorption<br>(trans. factors: 0.666 - 0.979)

C. Structure Solution and Refinement

Structure Solution	Direct Methods
Refinement	Full-matrix least-squares on F^2
Function Minimized	Σ w $(Fo^2 - Fc^2)^2$
Least Squares Weights	w = 1/ [$\sigma^2(Fo^2)$ + $(0.1157 \cdot P)^2$ + 0.0000 $\cdot$ P] where P = (Max$(Fo^2$,0) + $2Fc^2)/3$
$2\theta_{max}$ cutoff	55.0^o
Anomalous Dispersion	All non-hydrogen atoms
No. Observations (All reflections)	5869
No. Variables	254
Reflection/Parameter Ratio	23.11
Residuals: R1 (I>2.00σ(I))	0.1071
Residuals: R (All reflections)	0.2881
Residuals: wR2 (All reflections)	0.2824
Goodness of Fit Indicator	1.047
Flack Parameter (Friedel pairs = 2518)	-0.0(4)
Max Shift/Error in Final Cycle	0.000
Maximum peak in Final Diff. Map	0.21 $e^-/Å^3$
Minimum peak in Final Diff. Map	-0.25 $e^-/Å^3$

Table 1. Atomic coordinates and B_{iso}/B_{eq}

atom	x	y	z	B_{eq}
Si1	0.3360(3)	0.40186(6)	0.7179(4)	12.4(1)
O1	0.2768(4)	0.3611(1)	0.8116(5)	7.8(1)
O2	0.4566(4)	0.2564(1)	1.0368(5)	7.7(1)
N1	0.2439(6)	0.2267(2)	1.0353(6)	6.3(2)
C1	0.3412(6)	0.3212(2)	0.8343(8)	6.9(2)
C2	0.3415(9)	0.2963(2)	0.6858(9)	7.5(2)
C3	0.4480(8)	0.2788(3)	0.6181(9)	8.2(2)
C4	0.4407(7)	0.2538(3)	0.4672(8)	9.7(2)
C5	0.4621(7)	0.2063(3)	0.4977(8)	9.3(2)
C6	0.3389(7)	0.1864(3)	0.5752(8)	8.7(2)
C7	0.3635(9)	0.1408(3)	0.615(1)	11.1(3)
C8	0.2468(9)	0.1193(3)	0.710(1)	10.9(2)
C9	0.294(2)	0.0751(3)	0.753(2)	18.4(5)
C10	0.205(3)	0.0492(4)	0.760(3)	39(2)
C11	0.2113(6)	0.1449(2)	0.8570(9)	8.8(2)
C12	0.3326(6)	0.1565(2)	0.9613(8)	8.1(2)
C13	0.2892(6)	0.1860(2)	1.0896(7)	7.0(2)
C14	0.3292(5)	0.2590(2)	1.0135(6)	6.0(2)
C15	0.2626(5)	0.3002(2)	0.9716(7)	6.5(2)
C16	0.2564(7)	0.3279(2)	1.1154(8)	8.4(2)
C17	0.5272(7)	0.4050(3)	0.715(1)	11.6(3)
C18	0.279(2)	0.3935(5)	0.490(1)	20.5(6)
C19	0.237(1)	0.4437(3)	0.757(3)	20.7(7)
C20	0.270(1)	0.4840(2)	0.654(2)	19.8(5)
C21	0.079(1)	0.4354(3)	0.748(3)	22.8(8)
C22	0.323(3)	0.4505(5)	0.954(2)	29(1)

$B_{eq} = 8/3\ \pi^2(U_{11}(aa*)^2 + U_{22}(bb*)^2 + U_{33}(cc*)^2 + 2U_{12}(aa*bb*)\cos\gamma + 2U_{13}(aa*cc*)\cos\beta + 2U_{23}(bb*cc*)\cos\alpha)$

Table 2. Atomic coordinates and B_{iso} involving hydrogen atoms

atom	x	y	z	B_{iso}
H1	0.4392	0.3258	0.8663	8.27
H2	0.259(5)	0.290(2)	0.666(6)	5(2)
H3	0.543(9)	0.271(2)	0.69(1)	15(3)
H4A	0.5127	0.2638	0.3955	11.67
H4B	0.3497	0.2582	0.4181	11.67

H5A	0.4795	0.1921	0.3986	11.22
H5B	0.5446	0.2024	0.5633	11.22
H6A	0.2579	0.1886	0.5064	10.45
H6B	0.3175	0.2018	0.6707	10.45
H7A	0.4509	0.1386	0.6734	13.38
H7B	0.3758	0.1252	0.5179	13.38
H8	0.1621	0.1173	0.6448	13.04
H9A	0.3631	0.0659	0.6770	22.14
H9B	0.3405	0.0763	0.8545	22.14
H10A	0.2036	0.0332	0.6645	47.40
H10B	0.1152	0.0626	0.7750	47.40
H10C	0.2235	0.0306	0.8469	47.40
H11A	0.1650	0.1708	0.8243	10.59
H11B	0.1439	0.1289	0.9186	10.59
H12A	0.3716	0.1310	1.0071	9.74
H12B	0.4057	0.1697	0.8987	9.74
H13A	0.2131	0.1731	1.1486	8.46
H13B	0.3680	0.1898	1.1607	8.46
H15	0.1658	0.2946	0.9377	7.83
H16A	0.3502	0.3332	1.1525	10.05
H16B	0.2033	0.3139	1.1960	10.05
H16C	0.2117	0.3542	1.0894	10.05
H17A	0.5653	0.3787	0.6778	13.93
H17B	0.5612	0.4103	0.8194	13.93
H17C	0.5561	0.4275	0.6470	13.93
H18A	0.2174	0.4161	0.4580	24.56
H18B	0.2306	0.3670	0.4786	24.56
H18C	0.3614	0.3935	0.4246	24.56
H20A	0.1878	0.4917	0.5952	23.72
H20B	0.3459	0.4777	0.5836	23.72
H20C	0.2965	0.5070	0.7215	23.72
H21A	0.0433	0.4449	0.6485	27.42
H21B	0.0316	0.4503	0.8308	27.42
H21C	0.0612	0.4056	0.7589	27.42
H22A	0.2522	0.4557	1.0324	34.43
H22B	0.3871	0.4739	0.9505	34.43
H22C	0.3739	0.4252	0.9807	34.43
H23	0.173(5)	0.230(2)	1.010(5)	4(2)

Table 3. Anisotropic displacement parameters

atom	U_{11}	U_{22}	U_{33}	U_{12}	U_{13}

						U_{23}
Si1	0.108(2)	0.109(2)	0.254(4)	-0.003(2)	0.001(2)	0.071(2)
O1	0.095(3)	0.073(3)	0.127(4)	-0.003(2)	0.001(3)	0.019(2)
O2	0.047(2)	0.114(3)	0.131(4)	0.003(2)	-0.003(2)	0.016(3)
N1	0.045(3)	0.093(4)	0.100(4)	0.006(3)	0.002(3)	0.007(3)
C1	0.067(3)	0.080(4)	0.115(6)	-0.009(3)	-0.002(4)	0.014(4)
C2	0.090(5)	0.087(4)	0.107(6)	-0.013(4)	-0.030(5)	0.010(4)
C3	0.082(5)	0.139(6)	0.091(6)	0.006(5)	0.002(5)	0.015(5)
C4	0.104(5)	0.200(8)	0.066(5)	-0.021(5)	0.017(4)	-0.007(6)
C5	0.104(5)	0.157(7)	0.095(6)	0.005(5)	-0.001(5)	-0.011(5)
C6	0.096(5)	0.135(6)	0.100(5)	-0.000(5)	-0.012(4)	-0.024(4)
C7	0.148(7)	0.124(6)	0.152(8)	0.028(6)	-0.003(6)	-0.062(6)
C8	0.136(6)	0.124(6)	0.153(8)	-0.009(5)	0.013(6)	-0.031(6)
C9	0.31(2)	0.078(6)	0.31(2)	0.017(7)	0.03(2)	-0.043(9)
C10	0.82(5)	0.106(9)	0.58(4)	-0.03(2)	-0.26(4)	0.05(2)
C11	0.101(5)	0.094(5)	0.141(6)	-0.007(4)	-0.007(5)	-0.007(5)
C12	0.084(4)	0.098(4)	0.126(6)	0.017(4)	-0.016(4)	0.012(4)
C13	0.077(4)	0.087(4)	0.103(5)	0.017(3)	0.006(3)	0.011(4)
C14	0.049(3)	0.088(4)	0.089(5)	0.003(3)	0.007(3)	0.010(4)
C15	0.054(3)	0.085(4)	0.108(5)	-0.005(3)	0.001(4)	0.005(4)
C16	0.108(5)	0.104(5)	0.106(5)	0.009(4)	0.015(4)	-0.001(4)
C17	0.116(5)	0.122(5)	0.203(9)	-0.016(4)	-0.008(6)	

						0.032(6)
C18	0.33(2)	0.33(2)	0.118(8)	-0.13(2)	-0.09(1)	0.048(9)
C19	0.135(8)	0.098(6)	0.55(3)	0.011(6)	0.03(2)	0.09(1)
C20	0.200(9)	0.084(5)	0.47(2)	-0.005(6)	-0.03(2)	0.078(8)
C21	0.148(8)	0.138(7)	0.58(3)	0.044(6)	0.07(2)	0.07(2)
C22	0.62(4)	0.25(2)	0.23(2)	-0.12(2)	0.00(2)	-0.09(2)

The general temperature factor expression: $\exp(-2\pi^2(a^{*2}U_{11}h^2 + b^{*2}U_{22}k^2 + c^{*2}U_{33}l^2 + 2a^*b^*U_{12}hk + 2a^*c^*U_{13}hl + 2b^*c^*U_{23}kl))$

Table 4. Bond lengths (Å)

atom	atom	distance	atom	atom	distance
Si1	O1	1.618(5)	Si1	C17	1.817(7)
Si1	C18	2.038(10)	Si1	C19	1.660(10)
O1	C1	1.417(7)	O2	C14	1.228(6)
N1	C13	1.436(8)	N1	C14	1.316(7)
C1	C2	1.490(10)	C1	C15	1.538(9)
C2	C3	1.290(11)	C3	C4	1.512(11)
C4	C5	1.539(12)	C5	C6	1.483(10)
C6	C7	1.503(11)	C7	C8	1.532(12)
C8	C9	1.516(12)	C8	C11	1.528(11)
C9	C10	1.18(3)	C11	C12	1.500(9)
C12	C13	1.496(9)	C14	C15	1.494(8)

C15	C16	1.508(8)	C19	C20	1.579(17)
C19	C21	1.526(14)	C19	C22	1.88(3)

Table 5. Bond lengths involving hydrogens (Å)

atom	atom	distance	atom	atom	distance
N1	H23	0.71(5)	C1	H1	0.980
C2	H2	0.83(5)	C3	H3	1.11(9)
C4	H4A	0.970	C4	H4B	0.970
C5	H5A	0.970	C5	H5B	0.970
C6	H6A	0.970	C6	H6B	0.970
C7	H7A	0.970	C7	H7B	0.970
C8	H8	0.980	C9	H9A	0.970
C9	H9B	0.970	C10	H10A	0.960
C10	H10B	0.960	C10	H10C	0.960
C11	H11A	0.970	C11	H11B	0.970
C12	H12A	0.970	C12	H12B	0.970
C13	H13A	0.970	C13	H13B	0.970
C15	H15	0.980	C16	H16A	0.960
C16	H16B	0.960	C16	H16C	0.960
C17	H17A	0.960	C17	H17B	0.960
C17	H17C	0.960	C18	H18A	0.960
C18	H18B	0.960	C18	H18C	

					0.960
C20	H20A	0.960	C20	H20B	0.960
C20	H20C	0.960	C21	H21A	0.960
C21	H21B	0.960	C21	H21C	0.960
C22	H22A	0.960	C22	H22B	0.960
C22	H22C	0.960			

Table 6. Bond angles (^{o})

atom	atom	atom	angle	atom	atom	atom	angle
O1	Si1	C17	113.3(3)	O1	Si1	C18	106.0(5)
O1	Si1	C19	109.8(6)	C17	Si1	C18	105.1(5)
C17	Si1	C19	121.8(5)	C18	Si1	C19	98.3(8)
Si1	O1	C1	129.0(4)	C13	N1	C14	123.9(5)
O1	C1	C2	110.8(6)	O1	C1	C15	106.2(5)
C2	C1	C15	114.8(5)	C1	C2	C3	127.5(8)
C2	C3	C4	124.8(7)	C3	C4	C5	111.2(6)
C4	C5	C6	112.7(6)	C5	C6	C7	112.7(6)
C6	C7	C8	115.7(7)	C7	C8	C9	109.0(8)
C7	C8	C11	111.1(6)	C9	C8	C11	110.9(8)
C8	C9	C10	116.4(14)	C8	C11	C12	116.4(6)
C11	C12	C13	112.0(5)	N1	C13	C12	114.0(5)
O2	C14	N1	122.1(5)	O2	C14	C15	120.9(5)

N1	C14	C15	116.8(5)	C1	C15	C14	110.7(5)
C1	C15	C16	112.8(5)	C14	C15	C16	109.3(5)
Si1	C19	C20	114.7(11)	Si1	C19	C21	114.2(7)
Si1	C19	C22	91.3(9)	C20	C19	C21	108.0(10)
C20	C19	C22	108.5(9)	C21	C19	C22	119.6(15)

Table 7. Bond angles involving hydrogens (°)

atom	atom	atom	angle	atom	atom	atom	angle
C13	N1	H23	122(4)	C14	N1	H23	114(4)
O1	C1	H1	108.2	C2	C1	H1	108.3
C15	C1	H1	108.3	C1	C2	H2	108(4)
C3	C2	H2	122(4)	C2	C3	H3	120(5)
C4	C3	H3	112(4)	C3	C4	H4A	109.4
C3	C4	H4B	109.4	C5	C4	H4A	109.4
C5	C4	H4B	109.4	H4A	C4	H4B	108.0
C4	C5	H5A	109.1	C4	C5	H5B	109.1
C6	C5	H5A	109.1	C6	C5	H5B	109.1
H5A	C5	H5B	107.8	C5	C6	H6A	109.1
C5	C6	H6B	109.0	C7	C6	H6A	109.0
C7	C6	H6B	109.1	H6A	C6	H6B	107.8
C6	C7	H7A	108.4	C6	C7	H7B	108.4

C8	C7	H7A	108.4	C8	C7	H7B	108.4
H7A	C7	H7B	107.4	C7	C8	H8	108.6
C9	C8	H8	108.6	C11	C8	H8	108.6
C8	C9	H9A	108.2	C8	C9	H9B	108.2
C10	C9	H9A	108.2	C10	C9	H9B	108.2
H9A	C9	H9B	107.3	C9	C10	H10A	109.5
C9	C10	H10B	109.5	C9	C10	H10C	109.5
H10A	C10	H10B	109.5	H10A	C10	H10C	109.5
H10B	C10	H10C	109.5	C8	C11	H11A	108.2
C8	C11	H11B	108.2	C12	C11	H11A	108.2
C12	C11	H11B	108.2	H11A	C11	H11B	107.3
C11	C12	H12A	109.2	C11	C12	H12B	109.2
C13	C12	H12A	109.2	C13	C12	H12B	109.2
H12A	C12	H12B	107.9	N1	C13	H13A	108.8
N1	C13	H13B	108.8	C12	C13	H13A	108.8
C12	C13	H13B	108.8	H13A	C13	H13B	107.7
C1	C15	H15	108.0	C14	C15	H15	108.0
C16	C15	H15	108.0	C15	C16	H16A	109.5
C15	C16	H16B	109.5	C15	C16	H16C	109.5
H16A	C16	H16B	109.5	H16A	C16	H16C	109.5
H16B	C16	H16C	109.5	Si1	C17	H17A	109.5

Si1	C17	H17B	109.5	Si1	C17	H17C	109.5
H17A	C17	H17B	109.5	H17A	C17	H17C	109.5
H17B	C17	H17C	109.5	Si1	C18	H18A	109.5
Si1	C18	H18B	109.5	Si1	C18	H18C	109.5
H18A	C18	H18B	109.5	H18A	C18	H18C	109.5
H18B	C18	H18C	109.5	C19	C20	H20A	109.5
C19	C20	H20B	109.5	C19	C20	H20C	109.5
H20A	C20	H20B	109.5	H20A	C20	H20C	109.5
H20B	C20	H20C	109.5	C19	C21	H21A	109.5
C19	C21	H21B	109.5	C19	C21	H21C	109.5
H21A	C21	H21B	109.5	H21A	C21	H21C	109.5
H21B	C21	H21C	109.5	C19	C22	H22A	109.5
C19	C22	H22B	109.5	C19	C22	H22C	109.5
H22A	C22	H22B	109.5	H22A	C22	H22C	109.5
H22B	C22	H22C	109.5				

Table 8. Torsion Angles(°)
(Those having bond angles > 160 or < 20 degrees are excluded.)

atom1	atom2	atom3	atom4	angle	atom1	atom2	atom3	atom4	angle
C17	Si1	O1	C1	-28.1(6)	C18	Si1	O1	C1	86.7(6)
O1	Si1	C19	C20	-171.6(7)	O1	Si1	C19	C21	-46.2(14)
O1	Si1	C19	C22	77.3(4)	C19	Si1	O1	C1	-168.1(7)

C17	Si1	C19	C20	52.4(14)	C17	Si1	C19	C21	177.8(8)
C17	Si1	C19	C22	-58.7(9)	C18	Si1	C19	C20	-61.2(10)
C18	Si1	C19	C21	64.2(13)	C18	Si1	C19	C22	-172.3(5)
Si1	O1	C1	C2	-72.2(6)	Si1	O1	C1	C15	162.5(4)
C13	N1	C14	O2	1.0(9)	C13	N1	C14	C15	-174.2(5)
C14	N1	C13	C12	-88.9(6)	O1	C1	C2	C3	126.8(6)
O1	C1	C15	C14	-175.8(4)	O1	C1	C15	C16	-53.1(5)
C2	C1	C15	C14	61.4(6)	C2	C1	C15	C16	-175.9(5)
C15	C1	C2	C3	-112.8(7)	C1	C2	C3	C4	179.7(6)
C2	C3	C4	C5	-108.4(8)	C3	C4	C5	C6	72.4(7)
C4	C5	C6	C7	-176.1(5)	C5	C6	C7	C8	174.0(6)
C6	C7	C8	C9	-174.6(6)	C6	C7	C8	C11	-52.2(9)
C7	C8	C9	C10	-146.7(11)	C7	C8	C11	C12	-52.5(8)
C9	C8	C11	C12	68.8(9)	C11	C8	C9	C10	90.8(12)
C8	C11	C12	C13	172.7(5)	C11	C12	C13	N1	-65.3(6)
O2	C14	C15	C1	50.8(7)	O2	C14	C15	C16	-74.0(6)
N1	C14	C15	C1	-134.0(5)	N1	C14	C15	C16	101.2(5)

Table 9. Possible hydrogen bonds

Donor	H	Acceptor	D...A	D-H	H...A	D-H...A
N1	H23	O2[1]	2.846(6)	0.71	2.14(5)	173(5)

Symmetry Operators:

(1) X+1/2-1,-Y+1/2,-Z+2

Table 10. Intramolecular contacts less than 3.60 Å

atom	atom	distance	atom	atom	distance
Si1	C2	3.352(7)	O1	C3	3.485(9)
O1	C16	2.802(8)	O1	C21	3.060(10)
O1	C22	3.111(15)	O2	C1	2.896(7)
O2	C2	3.427(9)	O2	C12	3.435(7)
O2	C13	2.773(7)	O2	C16	3.032(7)
N1	C1	3.568(8)	N1	C11	3.017(8)
N1	C16	3.278(7)	C1	C17	3.345(9)
C2	C5	3.466(10)	C2	C14	3.036(9)
C2	C18	3.552(14)	C3	C6	3.123(11)
C3	C15	3.555(10)	C6	C11	2.994(10)
C6	C12	3.425(10)	C7	C10	3.494(19)
C7	C12	3.008(11)	C9	C12	3.149(13)
C10	C11	3.142(14)	C12	C14	3.274(8)
C17	C20	3.533(11)	C17	C22	3.16(2)
C18	C20	3.190(16)	C18	C21	3.197(19)

Table 11. Intramolecular contacts less than 3.60 Å involving hydrogens

atom	atom	distance	atom	atom	distance

Si1	H1	2.890	Si1	H20A	3.340
Si1	H20B	2.662	Si1	H20C	3.348
Si1	H21A	3.150	Si1	H21B	3.411
Si1	H21C	2.635	Si1	H22A	3.275
Si1	H22B	3.061	Si1	H22C	2.387
O1	H2	2.59(5)	O1	H15	2.588
O1	H16A	3.115	O1	H16C	2.458
O1	H17A	3.018	O1	H17B	3.117
O1	H18A	3.526	O1	H18B	2.878
O1	H18C	3.547	O1	H21C	2.525
O1	H22A	3.545	O1	H22C	2.655
O2	H1	2.641	O2	H3	3.12(9)
O2	H12B	3.023	O2	H13B	2.503
O2	H15	3.131	O2	H16A	2.814
O2	H16B	3.308	O2	H23	2.82(5)
N1	H6B	3.281	N1	H11A	2.631
N1	H11B	3.386	N1	H12A	3.272
N1	H12B	2.639	N1	H15	2.421
N1	H16B	3.105	C1	H3	2.79(8)
C1	H16A	2.740	C1	H16B	3.357
C1	H16C	2.708	C1	H17A	3.103

C1	H17B	3.513	C1	H18B	3.522
C1	H22C	3.536	C2	H4A	3.135
C2	H4B	2.583	C2	H6B	3.004
C2	H15	2.719	C2	H17A	3.365
C2	H18B	3.038	C3	H1	2.588
C3	H5A	3.334	C3	H5B	2.627
C3	H6A	3.510	C3	H6B	2.770
C3	H17A	3.393	C4	H2	2.67(5)
C4	H6A	2.717	C4	H6B	2.661
C5	H2	3.57(5)	C5	H3	2.72(8)
C5	H7A	2.615	C5	H7B	2.699
C6	H2	3.44(5)	C6	H3	3.44(8)
C6	H4A	3.326	C6	H4B	2.640
C6	H8	2.819	C6	H11A	2.735
C6	H12B	2.879	C7	H5A	2.694
C7	H5B	2.638	C7	H9A	2.427
C7	H9B	2.894	C7	H11A	2.764
C7	H11B	3.344	C7	H12A	3.358
C7	H12B	2.618	C8	H6A	2.798
C8	H6B	2.716	C8	H10A	2.785
C8	H10B	2.256	C8	H10C	3.049

C8	H12A	2.819	C8	H12B	2.720
C9	H7A	2.596	C9	H7B	2.675
C9	H11A	3.323	C9	H11B	2.629
C9	H12A	2.891	C9	H12B	3.412
C10	H7B	3.563	C10	H8	2.406
C10	H11B	2.921	C11	H6A	3.322
C11	H6B	2.604	C11	H7A	2.768
C11	H7B	3.345	C11	H9A	3.268
C11	H9B	2.494	C11	H10B	2.847
C11	H13A	2.640	C11	H13B	3.306
C11	H23	3.02(4)	C12	H6B	2.866
C12	H7A	2.757	C12	H8	3.381
C12	H9B	2.696	C12	H23	2.81(5)
C13	H11A	2.595	C13	H11B	2.699
C14	H1	2.673	C14	H2	3.19(5)
C14	H3	3.46(9)	C14	H6B	3.439
C14	H11A	3.580	C14	H12B	3.076
C14	H13A	3.151	C14	H13B	2.551
C14	H16A	2.641	C14	H16B	2.621
C14	H16C	3.279	C15	H2	2.63(5)
C15	H23	2.39(4)	C16	H1	2.743

C16	H22C	3.472	C16	H23	3.31(4)
C17	H1	2.939	C17	H18C	2.957
C17	H20B	3.086	C17	H22B	3.249
C17	H22C	2.766	C18	H17A	3.192
C18	H17C	3.144	C18	H20A	3.349
C18	H20B	2.856	C18	H21A	3.078
C18	H21C	3.112	C19	H17B	3.299
C19	H17C	3.215	C19	H18A	2.702
C19	H18B	3.397	C19	H18C	3.459
C20	H17C	3.252	C20	H18A	2.770
C20	H18C	3.576	C20	H21A	2.483
C20	H21B	2.919	C20	H21C	3.297
C20	H22A	3.349	C20	H22B	2.777
C20	H22C	3.489	C21	H18A	2.868
C21	H18B	3.470	C21	H20A	2.439
C21	H20B	3.194	C21	H20C	3.077
C21	H22A	3.002	C21	H22C	3.449
C22	H16C	3.425	C22	H17B	2.837
C22	H17C	3.505	C22	H20A	3.567
C22	H20B	3.283	C22	H20C	2.684
C22	H21B	2.960	C22	H21C	3.312

H1	H2	2.676	H1	H3	2.511
H1	H15	2.843	H1	H16A	2.592
H1	H16C	3.015	H1	H17A	2.612
H1	H17B	2.942	H1	H22C	3.352
H2	H3	2.76(10)	H2	H4A	3.430
H2	H4B	2.485	H2	H6A	3.473
H2	H6B	2.833	H2	H15	2.487
H2	H18B	2.937	H2	H23	3.58(7)
H3	H4A	2.515	H3	H4B	2.966
H3	H5A	3.557	H3	H5B	2.414
H3	H6B	3.065	H3	H17A	3.419
H4A	H5A	2.290	H4A	H5B	2.431
H4A	H6A	3.523	H4A	H6B	3.575
H4B	H5A	2.434	H4B	H5B	2.842
H4B	H6A	2.487	H4B	H6B	2.814
H5A	H6A	2.298	H5A	H6B	2.799
H5A	H7A	2.903	H5A	H7B	2.547
H5B	H6A	2.799	H5B	H6B	2.342
H5B	H7A	2.398	H5B	H7B	2.948
H5B	H12B	3.315	H6A	H7A	2.807
H6A	H7B	2.299	H6A	H8	2.702

H6A	H11A	2.905	H6B	H7A	2.367
H6B	H7B	2.808	H6B	H8	3.061
H6B	H11A	2.185	H6B	H11B	3.536
H6B	H12B	2.348	H6B	H23	3.324
H7A	H8	2.832	H7A	H9A	2.448
H7A	H9B	2.715	H7A	H11A	3.171
H7A	H11B	3.599	H7A	H12A	2.953
H7A	H12B	2.200	H7B	H8	2.312
H7B	H9A	2.318	H7B	H9B	3.277
H7B	H10A	3.566	H7B	H11A	3.593
H7B	H12B	3.551	H8	H9A	2.522
H8	H9B	2.783	H8	H10A	2.697
H8	H10B	2.105	H8	H10C	3.293
H8	H11A	2.282	H8	H11B	2.369
H8	H12B	3.575	H9A	H10A	1.837
H9A	H10B	2.499	H9A	H10C	2.259
H9A	H11B	3.542	H9A	H12A	3.488
H9B	H10A	2.484	H9B	H10B	2.285
H9B	H10C	1.826	H9B	H11A	3.433
H9B	H11B	2.560	H9B	H12A	2.185
H9B	H12B	3.044	H10B	H11A	3.482

H10B	H11B	2.445	H10C	H11B	3.261
H11A	H12A	2.804	H11A	H12B	2.372
H11A	H13A	2.802	H11A	H13B	3.507
H11A	H23	2.462	H11B	H12A	2.290
H11B	H12B	2.805	H11B	H13A	2.495
H11B	H13B	3.534	H11B	H23	3.314
H12A	H13A	2.344	H12A	H13B	2.275
H12B	H13A	2.809	H12B	H13B	2.349
H12B	H23	3.073	H13A	H23	2.195
H13B	H23	2.590	H15	H16A	2.813
H15	H16B	2.313	H15	H16C	2.329
H15	H23	2.127	H16A	H22C	3.266
H16B	H23	3.097	H16C	H21C	3.554
H16C	H22A	3.272	H16C	H22C	2.878
H17A	H18C	2.936	H17A	H22C	3.483
H17B	H20B	3.573	H17B	H22B	2.834
H17B	H22C	2.297	H17C	H18C	2.857
H17C	H20B	2.608	H17C	H20C	3.578
H17C	H22B	3.381	H17C	H22C	3.330
H18A	H20A	2.679	H18A	H20B	2.539
H18A	H21A	2.490	H18A	H21C	2.981

H18B	H21A	3.367	H18B	H21C	3.129
H18C	H20B	2.994	H20A	H21A	2.069
H20A	H21B	2.818	H20A	H21C	3.288
H20B	H21A	3.105	H20B	H22B	3.155
H20C	H21A	3.167	H20C	H21B	3.226
H20C	H22A	3.136	H20C	H22B	2.376
H20C	H22C	3.481	H21B	H22A	2.714
H21B	H22C	3.581	H21C	H22A	3.352
H21C	H22C	3.573			

Table 12. Intermolecular contacts less than 3.60 Å

atom	atom	distance	atom	atom	distance
O2	N1^{1}	2.846(6)	O2	C14^{1}	3.595(6)
O2	C15^{1}	3.412(6)	N1	O2^{2}	2.846(6)
C14	O2^{2}	3.595(6)	C15	O2^{2}	3.412(6)

Symmetry Operators:

(1) X+1/2,-Y+1/2,-Z+2 (2) X+1/2-1,-Y+1/2,-Z+2

Table 13. Intermolecular contacts less than 3.60 Å involving hydrogens

atom	atom	distance	atom	atom	distance
O2	H4A^{1}	3.113	O2	H4B^{1}	3.406
O2	H11A^{2}	3.260	O2	H15^{2}	2.566
O2	H23^{2}	2.14(5)	N1	H1^{3}	

	3.440			
N1	H3[3]	3.04(9)	N1	H4B[1]
	3.557			
C1	H13A[2]	3.537	C2	H5A[4]
	3.529			
C2	H5B[4]	3.528	C3	H6A[5]
	3.293			
C3	H13A[2]	3.551	C4	H2[5]
	3.51(5)			
C4	H6A[5]	3.528	C4	H13B[6]
	3.377			
C5	H2[5]	3.15(5)	C5	H13B[6]
	3.053			
C5	H16B[2]	3.531	C5	H18B[5]
	3.452			
C6	H4A[4]	3.483	C6	H13B[6]
	3.546			
C7	H18B[5]	3.583	C10	H22A[7]
	3.470			
C10	H22B[7]	3.537	C11	H16A[3]
	3.497			
C11	H17B[3]	3.563	C13	H1[3]
	3.364			
C13	H3[3]	3.31(8)	C13	H5A[1]
	3.200			
C13	H6A[1]	3.566	C13	H17A[3]
	3.556			
C14	H4B[1]	3.454	C14	H23[2]
	3.29(5)			
C16	H4B[1]	3.508	C16	H5B[3]
	3.530			
C16	H7A[3]	3.574	C16	H12B[3]
	3.331			
C16	H18B[1]	3.343	C16	H18C[1]
	3.500			
C17	H8[5]	3.399	C17	H11B[2]
	3.482			
C17	H13A[2]	3.251	C17	H20C[8]
	3.251			
C18	H7A[4]	3.557	C18	H16A[6]
	3.515			
C20	H10A[9]	3.143	C20	H17C[8]

					3.253
C21	H7B^{4}	3.537	C21	H12A^{3}	3.553
C22	H10C^{10}	3.082	H1	N1^{2}	3.440
H1	C13^{2}	3.364	H1	H11A^{2}	3.400
H1	H11B^{2}	3.032	H1	H13A^{2}	2.603
H1	H23^{2}	3.035	H2	C4^{4}	3.51(5)
H2	C5^{4}	3.15(5)	H2	H4A^{4}	2.931
H2	H5A^{4}	2.770	H2	H5B^{4}	2.831
H3	N1^{2}	3.04(9)	H3	C13^{2}	3.31(8)
H3	H4B^{5}	3.184	H3	H6A^{5}	2.921
H3	H13A^{2}	2.774	H3	H13B^{2}	3.569
H3	H16B^{2}	3.244	H3	H23^{2}	2.86(10)
H4A	O2^{6}	3.113	H4A	C6^{5}	3.483
H4A	H2^{5}	2.931	H4A	H6A^{5}	2.896
H4A	H6B^{5}	3.142	H4A	H11A^{5}	3.145
H4A	H13B^{6}	3.374	H4A	H16A^{6}	3.392
H4B	O2^{6}	3.406	H4B	N1^{6}	3.557
H4B	C14^{6}	3.454	H4B	C16^{6}	3.508
H4B	H3^{4}	3.184	H4B	H5B^{4}	3.156
H4B	H13B^{6}	3.089	H4B	H16A^{6}	3.281
H4B	H16B^{6}	2.936	H5A	C2^{5}	3.529
H5A	C13^{6}	3.200	H5A	H2^{5}	

Atom	Atom	Length/Å	Atom	Atom	Length/Å
					2.770
H5A	H13A^{6}	3.362	H5A	H13B^{6}	2.289
H5A	H15^{5}	3.394	H5A	H18B^{5}	3.206
H5A	H21C^{5}	3.461	H5B	C2^{5}	3.528
H5B	C16^{2}	3.530	H5B	H2^{5}	2.831
H5B	H4B^{5}	3.156	H5B	H16B^{2}	2.598
H5B	H18B^{5}	2.841	H6A	C3^{4}	3.293
H6A	C4^{4}	3.528	H6A	C13^{6}	3.566
H6A	H3^{4}	2.921	H6A	H4A^{4}	2.896
H6A	H13A^{6}	3.118	H6A	H13B^{6}	3.127
H6A	H17A^{4}	3.216	H6B	H4A^{4}	3.142
H7A	C16^{2}	3.574	H7A	C18^{5}	3.557
H7A	H16B^{2}	3.040	H7A	H16C^{2}	3.204
H7A	H18A^{5}	3.264	H7A	H18B^{5}	2.960
H7B	C21^{5}	3.537	H7B	H17A^{4}	3.389
H7B	H18A^{5}	3.501	H7B	H18B^{5}	3.377
H7B	H21A^{5}	3.075	H7B	H21C^{5}	3.100
H8	C17^{4}	3.399	H8	H17A^{4}	2.902
H8	H17C^{4}	3.035	H8	H18C^{4}	2.935
H9A	H18A^{5}	3.600	H9A	H20A^{11}	3.338
H9A	H21A^{5}	3.277	H9B	H21B^{2}	3.346
H10A	C20^{11}	3.143	H10A	H17C^{4}	

					3.249
H10A	H20A^{11}	2.774	H10A	H20B^{11}	2.789
H10A	H20C^{11}	3.394	H10A	H22A^{7}	3.587
H10B	H17B^{3}	3.599	H10B	H18C^{4}	3.260
H10B	H22B^{3}	3.391	H10B	H22C^{3}	3.119
H10C	C22^{7}	3.082	H10C	H22A^{7}	2.595
H10C	H22B^{7}	2.702	H11A	O2^{3}	3.260
H11A	H1^{3}	3.400	H11A	H4A^{4}	3.145
H11A	H16A^{3}	2.997	H11B	C17^{3}	3.482
H11B	H1^{3}	3.032	H11B	H16A^{3}	3.094
H11B	H17A^{3}	3.529	H11B	H17B^{3}	2.674
H11B	H22C^{3}	3.200	H12A	C21^{2}	3.553
H12A	H16C^{2}	3.364	H12A	H17B^{3}	3.547
H12A	H21B^{2}	3.292	H12A	H21C^{2}	2.925
H12B	C16^{2}	3.331	H12B	H15^{2}	3.052
H12B	H16B^{2}	2.983	H12B	H16C^{2}	3.002
H13A	C1^{3}	3.537	H13A	C3^{3}	3.551
H13A	C17^{3}	3.251	H13A	H1^{3}	2.603
H13A	H3^{3}	2.774	H13A	H5A^{1}	3.362
H13A	H6A^{1}	3.118	H13A	H17A^{3}	2.617
H13A	H17B^{3}	3.021	H13B	C4^{1}	3.377
H13B	C5^{1}	3.053	H13B	C6^{1}	

3.546

H13B	H3[3]	3.569	H13B	H4A[1]	3.374
H13B	H4B[1]	3.089	H13B	H5A[1]	2.289
H13B	H6A[1]	3.127	H13B	H15[2]	2.989
H13B	H21C[2]	3.598	H15	O2[3]	2.566
H15	H5A[4]	3.394	H15	H12B[3]	3.052
H15	H13B[3]	2.989	H16A	C11[2]	3.497
H16A	C18[1]	3.515	H16A	H4A[1]	3.392
H16A	H4B[1]	3.281	H16A	H11A[2]	2.997
H16A	H11B[2]	3.094	H16A	H18B[1]	3.187
H16A	H18C[1]	3.005	H16B	C5[3]	3.531
H16B	H3[3]	3.244	H16B	H4B[1]	2.936
H16B	H5B[3]	2.598	H16B	H7A[3]	3.040
H16B	H12B[3]	2.983	H16B	H18B[1]	2.949
H16B	H18C[1]	3.522	H16C	H7A[3]	3.204
H16C	H12A[3]	3.364	H16C	H12B[3]	3.002
H16C	H18B[1]	3.347	H16C	H18C[1]	3.425
H17A	C13[2]	3.556	H17A	H6A[5]	3.216
H17A	H7B[5]	3.389	H17A	H8[5]	2.902
H17A	H11B[2]	3.529	H17A	H13A[2]	2.617
H17B	C11[2]	3.563	H17B	H10B[2]	3.599
H17B	H11B[2]	2.674	H17B	H12A[2]	

					3.547
H17B	H13A[2]	3.021	H17B	H20C[8]	3.062
H17C	C20[8]	3.253	H17C	H8[5]	3.035
H17C	H10A[5]	3.249	H17C	H20A[8]	3.557
H17C	H20B[8]	3.186	H17C	H20C[8]	2.581
H18A	H7A[4]	3.264	H18A	H7B[4]	3.501
H18A	H9A[4]	3.600	H18B	C5[4]	3.452
H18B	C7[4]	3.583	H18B	C16[6]	3.343
H18B	H5A[4]	3.206	H18B	H5B[4]	2.841
H18B	H7A[4]	2.960	H18B	H7B[4]	3.377
H18B	H16A[6]	3.187	H18B	H16B[6]	2.949
H18B	H16C[6]	3.347	H18C	C16[6]	3.500
H18C	H8[5]	2.935	H18C	H10B[5]	3.260
H18C	H16A[6]	3.005	H18C	H16B[6]	3.522
H18C	H16C[6]	3.425	H20A	H9A[9]	3.338
H20A	H10A[9]	2.774	H20A	H17C[8]	3.557
H20A	H21A[12]	3.008	H20A	H21B[12]	3.426
H20B	H10A[9]	2.789	H20B	H17C[8]	3.186
H20B	H20B[8]	3.247	H20C	C17[8]	3.251
H20C	H10A[9]	3.394	H20C	H17B[8]	3.062
H20C	H17C[8]	2.581	H20C	H21B[12]	3.520
H21A	H7B[4]	3.075	H21A	H9A[4]	

					3.277
H21A	H20A^{12}	3.008	H21A	H21A^{12}	3.585
H21B	H9B^{3}	3.346	H21B	H12A^{3}	3.292
H21B	H20A^{12}	3.426	H21B	H20C^{12}	3.520
H21B	H21B^{12}	3.201	H21C	H5A^{4}	3.461
H21C	H7B^{4}	3.100	H21C	H12A^{3}	2.925
H21C	H13B^{3}	3.598	H22A	C10^{10}	3.470
H22A	H10A^{10}	3.587	H22A	H10C^{10}	2.595
H22B	C10^{10}	3.537	H22B	H10B^{2}	3.391
H22B	H10C^{10}	2.702	H22B	H22B^{8}	2.706
H22C	H10B^{2}	3.119	H22C	H11B^{2}	3.200
H23	O2^{3}	2.14(5)	H23	C14^{3}	3.29(5)
H23	H1^{3}	3.035	H23	H3^{3}	2.86(10)

Symmetry Operators:

(1) X,Y,Z+1
(2) X+1/2,-Y+1/2,-Z+2
(3) X+1/2-1,-Y+1/2,-Z+2
(4) X+1/2-1,-Y+1/2,-Z+1
(5) X+1/2,-Y+1/2,-Z+1
(6) X,Y,Z-1
(7) -X+1/2,Y+1/2-1,-Z+2
(8) -X+1,-Y+1,Z
(9) -X+1/2,Y+1/2,-Z+1
(10) -X+1/2,Y+1/2,-Z+2
(11) -X+1/2,Y+1/2-1,-Z+1
(12) -X,-Y+1,Z
